# Genetically-Guided Medical Nutrition Therapy in Type 2 Diabetes Mellitus and Pre-diabetes: A Series of *n*-of-1 Superiority Trials

**DOI:** 10.3389/fnut.2022.772243

**Published:** 2022-02-21

**Authors:** Kalliopi K. Gkouskou, Maria G. Grammatikopoulou, Evgenia Lazou, Despina Sanoudou, Dimitrios G. Goulis, Aristides G. Eliopoulos

**Affiliations:** ^1^Department of Biology, School of Medicine, National and Kapodistrian University of Athens, Athens, Greece; ^2^Embiodiagnostics Biology Research Company, Heraklion, Greece; ^3^Unit of Reproductive Endocrinology, First Department of Obstetrics and Gynecology, Faculty of Health Sciences, Medical School, Aristotle University of Thessaloniki, Thessaloniki, Greece; ^4^Department of Nutritional Sciences and Dietetics, Faculty of Health Sciences, International Hellenic University, Thessaloniki, Greece; ^5^Clinical Genomics and Pharmacogenomics Unit, Fourth Department of Internal Medicine, School of Medicine, National and Kapodistrian University of Athens, Athens, Greece; ^6^Center of Basic Research, Biomedical Research Foundation of the Academy of Athens, Athens, Greece

**Keywords:** nutrigenetics, T2DM (type 2 diabetes mellitus), obesity, precision nutrition, diet therapy, genetic risk score (GRS)

## Abstract

Type 2 diabetes mellitus (T2DM) is a heterogeneous metabolic disorder of multifactorial etiology that includes genetic and dietary influences. By addressing the latter, medical nutrition therapy (MNT) contributes to the management of T2DM or pre-diabetes toward achieving glycaemic control and improved insulin sensitivity. However, the clinical outcomes of MNT vary and may further benefit from personalized nutritional plans that take into consideration genetic variations associated with individual responses to macronutrients. The aim of the present series of *n*-of-1 trials was to assess the effects of genetically-guided vs. conventional MNT on patients with pre-diabetes or T2DM. A quasi-experimental, cross-over design was adopted in three Caucasian adult men with either diagnosis. Complete diet, bioclinical and anthropometric assessment was performed and a conventional MNT, based on the clinical practice guidelines was applied for 8 weeks. After a week of “wash-out,” a precision MNT was prescribed for an additional 8-week period, based on the genetic characteristics of each patient. Outcomes of interest included changes in body weight (BW), fasting plasma glucose (FPG), and blood pressure (BP). Collectively, the trials indicated improvements in BW, FPG, BP, and glycosylated hemoglobin (HbA1c) following the genetically-guided precision MNT intervention. Moreover, both patients with pre-diabetes experienced remission of the condition. We conclude that improved BW loss and glycemic control can be achieved in patients with pre-diabetes/T2DM, by coupling MNT to their genetic makeup, guiding optimal diet, macronutrient composition, exercise and oral nutrient supplementation in a personalized manner.

## Introduction

The prevalence and incidence of diabetes mellitus (DM) is increasing due to lifestyle shifts toward western-type diets and sedentary behaviors. There is, however, considerable heterogeneity in the association of dietary intake with the risk for developing type 2 diabetes mellitus (T2DM) and glycemic traits, which has been attributed to human genetic variation. Indeed, studies in twins have indicated a major contribution of genetics to the development of T2DM, which is comparable to the impact of diet (1). Genome-wide association studies (GWAS) have identified more than 140 genomic *loci* linked to increased T2DM predisposition (2) and individual meal responses (3). Several of these variants have been explored in combination to formulate genetic risk scores (GRS) to better predict the relative risk of T2DM development (4).

Medical nutrition therapy (MNT) is endorsed by both the Academy of Nutrition and Dietetics and the American Diabetes Association (ADA) for the management of adults with T2DM or pre-diabetes toward achieving euglycemia (5, 6). However, specific recommendations for the proportion of macronutrients, or oral nutrient supplementation (ONS) guidelines for the MNT of T2DM are lacking (6). As an example, the Obesity Society recommends a carbohydrate (CHO) intake between 45–65% of the energy intake (EI), 15–35% from protein, and 30% from total fat [10% from polyunsaturated fatty acid (PUFA), 15–20% from monounsaturated fatty acids (MUFA), 7% from saturated fatty acids (SFA)] for the management of T2DM (7). On the other hand, the Mediterranean diet, which confers significant benefits to T2DM prevention, is considered a high-fat diet (>40% of the EI from total fat), rich in MUFA but poor in SFA (8–10). This ambiguity in the MNT recommendations for patients with T2DM likely reflects the need to provide dietary and lifestyle advice personalized to the patient's bioclinical, phenotypic, lifestyle, and genetic data, thus maximizing its impact (11).

Several lines of clinical and experimental evidence call for a major conceptual and practical shift in dietetics toward the provision of precision nutrition care, parallel to the progress in precision medicine. Genetic variation is an important source of nutrition-related metabolic heterogeneity (12). Thus far, many observational studies have reported associations between specific genetic variants, predominantly single nucleotide polymorphisms (SNPs), and the health outcomes to the provision of specific macro- and micro-nutrients and ONS, including T2DM (11, 13, 14). However, how this information could be combined to provide dietary and lifestyle recommendations in T2DM remains poorly explored.

Since Hogben and Sim first conceived *n*-of-1 trials as components of pragmatic clinical practice in the 1950s (15), their application has gained residence. Today, the *n*-of-1 trial design represents the cornerstone of precision medicine and nutrition, offering insight on the individual patient response without requiring *post-hoc* analyses (16–19). The implementation of *n*-of-1 trials is used to compare interventions and find the most effective one for each patient, increasing treatment-response, reducing polypharmacy, minimizing adverse events, while conserving health care resources (20).

In recent years, nutrigenetic investigations have aided the delivery of MNT interventions in large-scale dietary trials, with the PREDIMED, the Look AHEAD and the POUNDS Lost clinical trials being notable examples. These trials reported milestone findings for the association of single SNPs with the outcome of MNT, including the interaction of a *TCF7L2* variant with a Mediterranean diet (MD) pattern for glycemic traits (21) and the interaction of a *MTNR1b* variant with dietary fat intake for glycemic traits (22–24). They have also identified associations between GRS for diabetes, and protein intake influencing insulin resistance (25). However, despite promising results, research and validations in different populations are still required. The conduction of nutritional *n*-of-1 trials may represent an advantageous method for gene-nutrition and diabetes-nutrition related research. Determination of the optimal personalized nutritional strategies, based on such trials is an essential step toward the delivery of precision prevention in the treatment of diabetes in the near future.

The present case series of *n*-of-1 cross-over superiority trials aimed to assess the effectiveness of precision, genetically guided MNT interventions vs. conventional MNT, in patients with pre-diabetes or T2DM.

## Materials and Methods

### Research Question, Trial Protocol, and Registration

The present series of *n*-of-1 cross-over, superiority trials is presented based on the standard protocol items recommendations for interventional trials (SPIRIT) extension for *n*-of-1 trial protocols (SPENT) statements (26). The protocol was registered at the Center for Open Sciences Framework (OSF) (https://bit.ly/3hmz3FJ). The research question based on the PICO acronym is detailed in Supplementary Table 1.

### Participants and Study Design

A quasi-experimental design was applied in three patients with pre-diabetes or T2DM (Supplementary Figure 1). A quasi-experimental design lacks patient randomization, and treatment allocation is dictated by the researchers. This design is the most appropriate one for a personalized nutrition intervention in which patients act as their own controls. Individuals provided consent to receiving personalized MNT based on their genetic profile, as well as for the anonymous publication of their data.

### Ethical Approval

Ethical approval for the study was obtained by the Bioethics Committee of the National and Kapodistrian University of Athens Medical School (1718034127/03-07-2018). Each participant provided consent prior to participation and additional consent for the collection and analysis of biological specimens. A detailed description of the methods is provided as Supplementary Material.

## Results

### Case 1

#### Patient Characteristics

A 45-year-old male patient with obesity, symptoms of hyperglycaemia (nocturia), suffering from night eating syndrome and fasting plasma glucose (FPG) 112 mg/dL reported involuntary body weight (BW) gain during the past 5 years despite exercising twice weekly, and difficulty in controlling BW and appetite since adolescence (Table 1). The patient also consumed branched-chain amino acid (BCAA) supplements, aiming at decreasing muscle soreness during resistance exercise sessions. The patient's main objectives were to reduce BW and maintain euglycemia.

**Table 1:** Patient characteristics at baseline.

			**Patient 1**	**Patient 2**	**Patient 3**
Diagnosis			Pre-diabetes	T2DM	Pre-diabetes
Occupation			IT expert	Businessman	Retiree
Age (years)			45	54	69
Ethnicity			Caucasian	Caucasian	Caucasian
Sex			Male	Male	Male
Exercise	Frequency		3–4 times/week	3–4 times/week	1 h walk/day
	Type		Mainly resistance	Combined endurance and resistance	Walking
Anthropometric indices	Body weight (kg)		105	82	72
	Height (cm)		182	177	175
	BMI (kg/m^2^)		31.5	26.2	23.5
	Waist circumference (cm)		104	80	71
	Hips circumference (cm)		90	82	73
	Weight status diagnosis		Obesity	Overweight	Normoweight
	Abdominal obesity diagnosis		yes	no	no
	SBP/DBP (mm Hg)		119/78	143/86	127/72
Lab results		Reference range^†^			
	FPG (mg/dL)	65–109 mg/dL	112	155	120
	GT at 2 h (mg/dL)^‡^		145	188	148
	HbA_1c_ (%)*		5.9	6.3	5.6
	Total cholesterol (mg/dL)	<200 mg/dL	192	230	188
	HDL-cholesterol (mg/dL)	≥40 mg/dL	43	49	43
	LDL-cholesterol (mg/dL)	<100 mg/dL	84	115	84
	Total cholesterol/HDL ratio		4.46	4.69	4.37
	Triglycerides (mg/dL)	<150 mg/dL	235	238	200
	25(OH)D (ng/dL)	20–40 ng/mL	19	21	19
	Ferritin (ng/dL)	20–250 ng/mL	40	50	25
Medications			–	Pitavastatin (2 mg/day), metformin (1,000 mg/day)	–
Oral nutrient supplementation			BCAA 5 g/day	–	–

BCAA, branched-chain amino-acids; BMI, body mass index; DBP, diastolic blood pressure; FPG, fasting plasma glucose; HbA_1c_, glycosylated hemoglobin; HDL, high-density lipoprotein; LDL, low-density lipoprotein; information technology; OGTT, oral glucose tolerance test; SBP, systolic blood pressure; T2DM, type 2 diabetes mellitus; WHR, waist-to-hips ratio; 25(OH)D, 25-hydroxy vitamin D; *HbA_1c_ ≥ 6.5%, T2DM diagnostic criterion; HbA_1c_ 5.9–6.4%, pre-diabetes diagnostic criterion; †for adult males;^‡^ <100 mg/dL, normal glucose metabolism; 100–125 mg/dL, impaired fasting glucose; >126 mg/dL, elevated hyperglycaemia.

#### Conventional Dietary Intervention

A diet high in fiber and carbohydrates and low in protein was initially prescribed by a registered dietician, divided into three meals and an equal number of snacks, aiming to reduce postprandial glucose peaks and prolonged fasting intervals (Table 2) (6). He was also prescribed vitamin D ONS to improve low serum vitamin D levels. Adherence to this dietary plan for 8 weeks reduced BW by 3 kg (2.9%) without altering his weight status. No change was noted in the HbA_1c_ levels and a marginal improvement in the FPG (106 mg/dL) and systolic blood pressure (BP) was achieved.

**Table 2:** Initial MNT prescribed to each case, and genetically-guided MNT prescribed post-nutrigenetic testing.

	**Patient 1**		**Patient 2**		**Patient 3**	
	**Initial MNT**	**Genetically-guided MNT**	**Initial MNT**	**Genetically-guided MNT**	**Initial MNT**	**Genetically-guided MNT**
EI (kcal/day)	Normocaloric (2,200)	Normocaloric (2,200)	Normocaloric (1,800)	Normocaloric (2,000)	Normocaloric (1,750)	Normocaloric (1,800)
Fiber (g)	33	32	33	25	33	26
Macronutrient distribution to the EI (%)	CHO: 50% Fat: 32% Protein: 18% MUFA: 15% PUFA: 10%	CHO: 49% Fat: 26% Protein: 25%	CHO: 50% Fat: 20% Protein: 30%	CHO: 45% Fat: 35% Protein: 20%	CHO: 45% Fat: 30% Protein: 25%	CHO: 47% Fat: 37% Protein: 16% high-n-3/low-n-6 PUFA
SFA distribution to the EI (%)	7%	No change	7%	9%	7%	No change
Dietary pattern						Greek-Mediterranean Diet
ONS	Vitamin D	Stop BCAA Avoid melatonin	None	Riboflavin (1.6 mg/d) for 16 weeks (27) Folate (5 mg/d) (28)	None	Zn (14 mg/d) (an average daily Zn intake of 14 mg can be achieved through ONS (~10 mg Zn) plus an average serv. of seafood/red meat/fish, or 3 serv of dairy (~2 cups of yogurt/milk and 3 ounces of cheese)
Ideal meal timing	1st meal: 7 a.m. 1st snack: 12 a.m. 2nd meal: 5 p.m. 2nd snack: 7 p.m. 3rd meal: 9:30 p.m. (avoid high CHO intake in the evening) 3rd meal: 2 a.m.	1st meal: 10:30 a.m. 1st snack: 1 a.m. 2nd snack: 4 p.m. 2nd meal: 7 p.m. 3rd snack: preferably during daylight (based on genetic makeup)	1st meal: 8 a.m. 1st snack: 11 a.m. 2st meal: 1:30 p.m. 2nd snack: 4 p.m. 3rd meal: 7 p.m. 3rd snack: 9:30 p.m.	No changes proposed	1st meal: 8 a.m. 1st snack: 11 a.m. 2nd meal: 1:30 p.m. 2nd snack: 4 p.m. 3rd meal: 7 p.m. 3rd snack: 9:30 p.m.	No changes proposed
Exercise type	Resistance exercise	Endurance exercise	Combination of resistance and endurance exercise	No changes proposed	Combination of resistance and endurance exercise	No changes proposed
Foods to consume or avoid	Increased fiber intake	No further changes proposed	Consume fiber for improved T2DM regulation	•Consume fiber for BW regulation •Avoid desserts •Avoid milk	Increased fiber intake	•Consume fiber for improved T2DM regulation •Consume fish/seafood and seeds

*BCAA, branched-chain amino-acids; BW, body weight; EI, energy intake; MNT, medical nutrition therapy; CHO, carbohydrate; ONS, oral nutrient supplementation; serv, serving; SFA, saturated fatty acids; PUFA, poly-unsaturated fatty acids; T2DM, type 2 diabetes mellitus*.

#### Personalized Lifestyle Intervention

A new dietary intervention was initiated in our Unit (Table 2), based on the results of a nutrigenetic test (11) (Table 3). The most relevant genetic information extracted from this analysis included: (i) a high GRS for T2DM, indicating that the patient could particularly benefit from a high-protein diet in improving insulin resistance (IR) and β-cell function (25); (ii) a high GRS for habitual coffee consumption which is linked to improved glycaemic responses to a low-fat diet (29), and (iii) a high GRS for FPG, associated with improved glucose metabolism when consuming low-fat diets.

**Table 3:** Individual polymorphisms identified in each case study, based on the nutrigenetic test.

		**Patient 1**	**Patient 2**	**Patient 3**
Polymorphisms associated with carbohydrate distribution to the EI	*MTNR1B* rs1387153 (C/T) T: risk allele for T2DM C: common allele	TT	CC	CC
	*APOA5* rs662799 (T/C) C: risk allele for dyslipidaemia T: common allele	TT	TT	TT
	*CRY1* rs2287161 (G/C minus) C: risk allele for mood disorders G: common allele	GC	CC	GC
	*PCSK7* rs236918 (C/G minus) G: risk allele (rare) for liver cirrhosis and high levels of ferritin, sTfR C: common allele	CC	CC	CC
	*GIPR* rs2287019 (C/T) C: risk allele for T2DM T: rare	CC	CT	TT
	*IRS1* rs2943641 (C/T) C: risk allele for T2DM T: rare	CT	CT	CT
	*PLIN-1* rs894160 (G/A minus) A: risk allele for increased waist circumference and T2DM G: common allele	GG	GG	GG
Polymorphisms checked for ideal fat distribution to the EI	**GRS** of 8 SNPs related to habitual coffee intake	high	Low	low
	**GRS** of 14 SNPs related to FPG	high	moderate	low
	*PPM1K* rs1440581 (C/T minus) C: risk allele for T2DM and high BCAA/AAA ratio T: common allele	CC	TT	TT
Polymorphisms for ideal protein distribution	**GRS** related to DM (31 SNPs)	high for DM	moderate for DM	low for DM
	**GRS** of 32SNPs related to BMI, WHR and T2DM	high for OB	low for OB	low for OB
	*DHCR7* rs12785878 (T/G) T: risk allele for vitamin D deficiency G: rare in Caucasians (no health effect)	TT	CT	CT
Polymorphisms associated with ideal MUFA, PUFA or SFA distribution	*IRS1* rs2943641 (C/T) C: risk allele for T2DM T: rare	CT	CT	CT
	*GIPR* rs2287019 (C/T) C: risk allele for T2DM T: rare	CT	CT	CT
	*PLIN-1* rs894160 (G/A minus) A: risk allele for increased waist circumference and T2DM G: common allele	GG	GG	GG
	*CLOCK* rs4580704 (G/C) G: protective effect for T2DM C: common allele	CC	CC	CC
	*CLOCK* rs1801260 (C/T) C: risk allele for MetS T: common allele	TT	TT	TT
	*TCF7L2* rs7903146 (C/T) T: risk allele for T2DM C: common allele	CT	TT	CC
	*TCF7L2* rs12255372, (G/T) T: risk allele for T2DM G: common allele	GG	GG	GG
	*LEPR* rs3790433 (G/A minus) G: risk allele (common) for IR A: rare allele	GA	GA	GG
Polymorphisms for ideal fiber intake	*TCF7L2* rs7903146 (C/T) T: risk allele for T2DM C: common allele	CT	TT	CC
	*GCKR* rs780094 (G/A minus) A: risk allele for T2DM and dyslipidaemia G: common allele	GG	AG	GG
	*GIPR* rs2287019 (C/T) C: risk allele for T2DM T: allele rare	CC	CT	CT
	*TCF7L2* rs12255372, (G/T) T: risk allele for T2DM G: common allele	GG	GT	GG
Specific foods to consume/avoid based on genotype	*TCF7L2* rs7903146 (C/T) T: risk allele for T2DM C: common allele	CT	TT	CC
SNPs/GRS associated with need for micronutrient supplementation in T2DM	*MTHFR* rs1801133 (C/T minus) T: risk allele for hypertension, T2DM, folate deficiency, and CVD C: common allele	CT	TT	CC
	*MTNR1B* rs10830963 (C/G) G: risk allele for T2DM C: common allele	GG	CC	CC
	*SLC30A8* rs13266634 C: risk allele T: rare, protective	CC	CT	TT
	*IRS1 rs2943641 (C/T)* C: risk allele for T2DM T: rare	CC	CC	CT
SNPs/GRS associated with lifestyle changes and exercise for T2DM management	*MTNR1B* rs1387153 (C/T) T: risk allele for T2DM C: common allele	TT	CC	CC
	**GRS** of 32SNPs related to BMI, WHR, and T2DM	high	low	low
	*SLC30A8* rs13266634 C: risk allele T: rare, protective	CC	CT	TT
	*PPARG* rs1801282 C: common G: rare, informative for exercise (30)	CG	CG	CG
SNPs/GRS linked to proper food timing for T2DM management	*MTNR1B* rs10830963 (G/C) G: risk allele for T2DM C: common allele	GG	GC	CC

*AAA, aromatic amino-acids; BCAA, branched-chain amino-acids; BMI, body mass index; CVD, cardiovascular disease; DBP, diastolic blood pressure; FPG, fasting plasma glucose; GRS: genetic risk score; HbA_1c_, glycosylated hemoglobin; HDL, high-density lipoprotein; LDL, low-density lipoprotein; MetS, metabolic syndrome; OB, obesity; OGTT, oral glucose tolerance test; SBP, systolic blood pressure; SNP, single-nucleotide polymorphism; sTfR, soluble transferrin receptor; T2DM, type diabetes mellitus; WHR, waist-to-hips ratio; 25(OH)D, 25-hydroxy vitamin D*.

Informative SNPs for macronutrient intake included *DHCR7* rs12785878, *PPM1K* rs1440581, and *MTNR1B* rs1387153, which further pointed to the putative beneficial effects of a low-calorie, low-fat and low-carbohydrate diet (11). The *MTNR1B* rs10830963 GG genotype (which is in linkage disequilibrium with rs1387153 in Greeks) was used to implement lifestyle and food-timing changes, as G homozygotes exhibit higher early melatonin onset during the evening, a longer duration of elevated melatonin concentrations and a delayed melatonin decline in the morning (31), the latter being associated with an increased risk for DM upon early waking up (32). Furthermore, the concurrence of meal timing with elevated endogenous melatonin is associated with impaired glucose tolerance in GG homozygotes (33). As a result, people carrying this variant display worse glucose tolerance when consuming dinner late when melatonin levels are high (34). Moreover, because of the prolonged duration of elevated melatonin levels, risk allele carriers may show a slower decline in melatonin concentrations during the morning hours; thus, delaying breakfast might reduce the risk of developing T2DM (35). These observations underpin opportunities for behavioral changes regarding the timing of food intake and hence specific recommendations for the optimal timing of breakfast and dinner were provided to the patient.

The patient was advised to avoid melatonin supplementation as prescription of melatonin to rs10830963 GG carriers is not linked to beneficial health outcomes (35, 36) (Table 3). In the absence of genetic information precluding vitamin D ONS, the patient continued its use. Considering that individuals genetically prone to obesity appear to benefit less from resistance exercise (37) and CC homozygotes for *SLC30A8* rs13266634 are more susceptible to soreness during resistance training (38), the patient was advised to replace resistance exercise with endurance training. Adherence to this diet and exercise intervention for 8 weeks achieved a 7.5 kg BW loss (7.4%) and a notable improvement in FPG levels (89 mg/dL; Table 4). Regarding metabolic control, improvements were noted in both the HbA_1c_ and FPG concentrations, as well as in all lipid parameters. Moreover, the reduction in BW resulted in remission of pre-diabetes (39, 40). Diastolic BP was marginally improved following the genetically-guided MNT interventions.

**Table 4:** End-of-treatment results and % of change for each endpoint and cases after implementation of the conventional (1st) and precision (2nd) MNT intervention periods.

		**Patient 1**				**Patient 2**				**Patient 3**			
		**1st period** **conventional MNT**		**2nd period** **precision MNT**		**1st period** **conventional MNT**		**2nd period** **precision MNT**		**1st period** **conventional MNT**		**2nd period** **precision MNT**	
		**Absolute**	***%Δ***	**Absolute**	***%Δ***	**Absolute**	***%Δ***	**Absolute**	***%Δ***	**Absolute**	***%Δ***	**Absolute**	***%Δ***
Diagnosis (related to T2DM)		–		–		T2DM		T2DM		–		–	
Pre-diabetes remission		–		√		N/A	N/A	√		√	
Body weight (kg)		102	*−2.9*	94.5	*−7.4*	78	*−4.9*	76	*−2.6*	72	*0*	71	*−1.4*
(kg/m^2^)		30.79	*−2.3*	28.53	*−7.3*	24.9	*−5*	24.26	*−2.6*	23.51	*0*	23.18	*−1.39*
Weight status		Obesity		Overweight		Normoweight		Normoweight		Normoweight		Normoweight	
Reduction in weight status tier		–		√		√		√		–		–	
WC (cm)		102	*−1.9*	98	*−3.9*	76	*−5*	75	*−1.4*	71	*0*	71	*0*
Abdominal obesity		√		√		–		–		–		–	
SBP/DBP (mm Hg)		115/78	*−3.4/0*	115/75	*0/−3.8*	140/85	*−2.1/−1.2*	128/80	*−8.6/−5.9*	127/72	*0*	125/71	*−1.6/−1.4*
Lab Results	Reference^†^												
FPG (mg/dL)	65–109	106	*−5.4*	89	*−16*	145	*−6.5*	108	*−25.5*	120	*0*	112	*−6.7*
OGTT at 2 h (mg/dL) ‡		138	*−4.8*	105	*−23.9*	182	*−3.2*	145	*−20.3*	148	*0*	132	*−10.8*
HbA_1c_ (%)*		5.9	*0*	5.4	*−8.5*	6.3	*0.0*	6	*−4.8*	5.6	*0*	5.4	*−3.6*
Total cholesterol (mg/dL)	<200	190	*1.0*	180	*−5.3*	210	*−8.7*	200	*−4.8*	190	*1.1*	188	*−1.1*
HDL–cholesterol (mg/dL)	≥40	44	*2.3*	46	*4.5*	49	*0*	49	*0*	40	*−7*	37	*−7.5*
LDL–cholesterol (mg/dL)	<100	84	*0*	78	*−7.1*	115	*0*	109	*−5.2*	84	*0*	84	*0*
Total cholesterol/HDL		4.31	*−3.3*	3.91	*−9.4*	4.29	*−8.6*	4.08	*−4.8*	5.1	*17.6*	4.4	*−14.9*
Triglycerides (mg/dL)	<150	235	*0*	210	*−10.6*	230	*−3.4*	230	*0*	200	*0*	200	*0*
25(OH)D (ng/dL)	20–40	28	*47.4*	30	*7.1*	24	*14.3*	25	*4.2*	25	*31.5*	33	*32*
Ferritin (ng/dL)	20–250	40	*0*	40	*0*	50	*0*	50	*0*	25	*0*	28	*12*
Adverse events (*n*)		0		0		0		0		0		0	

Δ, change; BMI, body mass index; DBP, diastolic blood pressure; FPG, fasting plasma glucose; HbA_1c_, glycosylated haemoglobin; HDL, high-density lipoprotein; LDL, low-density lipoprotein; N/A, not applicable; OGTT, oral glucose tolerance test; SBP, systolic blood pressure; T2DM, type 2 diabetes mellitus; WHR, waist-to-hips ratio; 25(OH)D, 25-hydroxy vitamin D; *HbA_1c_ ≥ 6.5%, T2DM diagnostic criterion; HbA_1c_ 5.9–6.4%, pre-diabetes diagnostic criterion; †for adult males;^‡^ <100 mg/dL, normal glucose metabolism; 100–125 mg/dL, impaired fasting glucose; >126 mg/dL, elevated hyperglycaemia. The %Δ is presented in italics.

### Case 2

#### Patient Characteristics

A 54-year-old male patient with overweight and T2DM (Table 1) aimed at reducing FPG (from 155 mg/dL), BW, serum triglycerides and cholesterol levels, and to control blood pressure through lifestyle changes. The patient reported exercising four times per week and claimed to be very careful with his nutritional choices.

#### Conventional Dietary Intervention

A normocaloric (2,000 kcal/day), high-fiber diet (30 g/day), with the EI divided in CHO (50%), fats (20%), and proteins (30%), was prescribed in three main meals and an equal number of snacks daily (Table 2). Food timing and exercise regimes were adjusted to his everyday schedule. Following 8 weeks of MNT, BW was reduced by 4 kg (4.9%), altering the weight status of the patient to normoweight. The intervention failed to induce any change in the HbA_1c_ concentrations and resulted in a marginal improvement in the FPG (145 mg/dL) and BP levels (Table 4).

#### Personalized Lifestyle Intervention

A new dietary intervention pattern was designed based on the patient's genetic profile (Tables 2, 3). The most nutritionally relevant genetic information included: (i) a low GRS for habitual coffee consumption (associated with beneficial health outcomes following a high-fat diet); (ii) the *PPM1K* rs1440581 TT genotype associated with a reduction in insulin and the β-cell function homeostatic model assessment (HOMA-B) following a high-fat diet (41), (iii) homozygosity of the risk C allele of *CRY1* rs2287161, linked with an impaired glycemic control following high-carbohydrate consumption (42); and (iv) homozygosity of the T allele of *TCF7L2* rs7903146, associated with a greater risk for T2DM and related comorbidities when consuming diets rich in saturated fatty acids (SFA ≥15.5% of EI) (43) desserts and milk (44).

Concerning ONS, the most informative SNP was *MTHFR* rs1801133, as administration of riboflavin supplements to TT homozygotes suffering from hypertension can improve blood pressure (BP) more efficiently than state-of-the-art antihypertensive drugs (45). Furthermore, due to the *MTHFR* homozygosity, the patient was asked to test blood concentrations of homocysteine (46) which were found to be elevated and the patient's dietary plan was supplemented with folate (0.5 mg/day) (28). Based on the genetic information, personalized dietary advice was provided as outlined on Table 2. Following 8 weeks of adhering to this intervention, an additional BW loss of 2 kg was achieved (2.6% in 2 months), coupled with a notable improvement in FPG (108 mg/dL) and BP (−12 mm/Hg) levels (Table 4).

### Case 3

#### Patient Characteristics

A 67-year-old, normal-weight male with T2DM experienced dysregulated FPG levels (122 mg/dL) (Table 1). Moreover, the patient reported experiencing depressive symptoms which were verified by the Beck Depression Inventory (BDI) (47) score of 15.

#### Conventional Dietary Intervention

A normocaloric (1,750 kcal/day), high-fiber (30 g) diet was initially prescribed (Table 2), divided into three meals and an equal number of snacks, to avoid postprandial glucose peaks and prolonged fasting intervals. ONS with vitamin D was also recommended. After 8 weeks of MNT, no improvements were detected in the BW, BP, HbA_1c_, or FPG concentrations (Table 4).

#### Personalized Lifestyle Intervention

A new dietary intervention was designed based on the patient's genetic profile (Table 3). The most informative scores related to macronutrient intake included: (i) low GRS for T2DM, for which low-protein diets have been shown to improve outcomes (25), (ii) low GRS for habitual coffee intake which has been associated with improved health following a high-fat diet (29), and (iii) low GRS for FPG, for which a high-fat diet may improve glucose metabolism (48). This patient was also carrier of the *LEPR* rs3790433 GG genotype which is associated with reduced risk for hyperinsulinemia and IR upon adherence to high-n-3/low-n-6 polyunsaturated fatty acid (PUFA) diet. The *SLC30A8* rs13266634 SNP was informative in relation to zinc supplementation which has been reported to particularly benefit those carrying no risk alleles (49).

No other usable genetic information was recorded with respect to carbohydrate intake, lifestyle changes, and exercise. Based on this genetic data, a high-fat traditional Cretan-Greek MD was prescribed (Table 2) that is enriched in n-3 PUFA (50, 51).

Despite the patient being normoweight from the start of the interventions, after 8 weeks of adhering to this personalized MNT program (Table 4), improvements were noted in the glycaemic profile (FPG 112 mg/dL and HbA_1c_ of 5.4%) resulting in the remission of pre-diabetes. Moreover, a significant improvement was noted in the subjective depressive symptoms reported by the patient, identified by a 10-point reduction in the BDI scale and a small improvement was recorded regarding the BP levels. No adverse events were reported regarding the conventional or precision interventions by either patient.

## Discussion

The horizontal approach to the provision of dietary recommendations has been questioned (52) as it often fails to address particularities at the individual patient level. Approximately 70 years ago, Williams (53) was the first to envisage personalized nutritional recommendations that take into account “genetic patterns” and suggested that patients are “far from standardized specimens.” Although this concept remained dormant for several decades, the potential of precision nutrition in health and disease management is nowadays increasingly recognized (11, 13, 14, 54) and corroborated by several observational studies linking dietary outcomes to genetic variants (55–59). Along these lines, results from the Diabetes Prevention Program have suggested that, although a standardized intensive lifestyle intervention reduces DM risk (60), its efficacy varies across individuals (61), and is determined by genetic factors (62).

Conceptually, the series of *n*-of-1 trials presented herein addresses the reverse. It explores combinations of genetic variants associated with responses to dietary and lifestyle parameters to provide personalized dietary plans for patients with T2DM or pre-diabetes. We applied a quasi-experimental *n*-of-1 trials design as the most appropriate to address the effectiveness of conventional vs. genotype-based MNT treatment in the same patient. This methodology has been successfully explored in patient-centered medicine and nutrition studies (18, 63, 64), including DM (65), and can form a pragmatic research extension of the standard care.

The results provide proof-of-principle that we can achieve better glycaemic control in T2DM patients by coupling MNT to genetic information as a predictor of optimal macronutrient composition and ONS in a personalized manner. Both patients with pre-diabetes experienced remission of the condition. We particularly note that the successful outcome of these interventions entailed very different macronutrient compositions, ranging from a high-fat traditional Mediterranean dietary pattern to a diet with greater-protein intake. We thus propose that genetically-guided MNT may in fact aid in the resolution of the “ambiguity” regarding the ideal macronutrient composition for the management of DM (6–8). Furthermore, opting for genetically-informed dietary recommendations may enhance patient adherence. For example, informing the second patient of his genetic susceptibility to increased risk for T2DM at high BCAA intake led to voluntary changes in lifestyle choices toward abandoning BCAA ONS that he was unwilling to do otherwise. Thus, adherence to genetically-guided lifestyle recommendations is likely to amplify health outcomes. Indeed, several surveys conclude that individuals are more likely to adhere to dietary recommendations if these are based on their genetic profile (66, 67), while, on the other hand, improving adherence to healthy dietary patterns could attenuate the genetic predisposition for weight gain (57).

Despite the complexity of gene-nutrient interactions, genetic (GRS) or polygenic risk scores (PRS) coupled with mathematical predictive models and machine-learning algorithms are expected to accelerate the translation of genetic information to nutritional recommendations (68) and to have a major impact on the practical applications of nutrigenetics to precision nutrition (13). Results presented herein support this notion and confirm the utility of GRS in guiding nutritional recommendations in T2DM. It could be argued that the GRS used herein have not been adjusted for ancestry and this may indeed be a limitation of our study. Nevertheless, our results confirm the utility of GRS in guiding nutritional recommendations in T2DM in Greek similar to other populations (25, 46).

Other limitations of the present study include the possible carryover effect of the first intervention cycle which was addressed by testing for treatment-by-period interaction, as suggested for nutrition clinical trials (69). We note that a rule of thumb for the ideal duration of the wash-out period in dietary interventions does not exist, with the exception of ONS trials (70). Nevertheless, according to Senn (71), performing analyses for assessing the carryover effect of cross-over trials is not advised and analyzing data according to treatment-by-period interaction is the preferred strategy. In this manner, the Cochrane handbook suggests that although long wash-out periods can avoid the carryover effect, this is not deemed essential (72). Wash-out periods should also take into consideration ethical aspects related to maintenance of MNT in T2DM patients, including the violation of the equipoise principle (73–75). Of note, the importance of a wash-out period separating treatment phases in *n*-of-1 trials has also been debated (76). According to the DEcIDE methods center *N*-of-1 guidance Panel (20), often, the benefits of an intervention wash-out quickly, but the risks of adverse events persist.

An additional limitation of the present series may involve the small number of participants. However, the *n*-of-1 design is unanimously the most appropriate one for the implementation of precision medicine/nutrition interventions in the pragmatic setting (77, 78). As per Mizra and Guyatt (79), for the design to be suitable, the disease course must involve a chronic and relatively stable condition, such as in T2DM. A possible bottleneck in this design may involve the higher probability for a type-I error, however, aggregation of *n*-of-1 trials appears to outperform traditional RCTs in many methodological domains (80). According to Kravitz et al. (81), the importance of precision medicine stems from the individual heterogeneity in the treatment effect, thus *n*-of-1 trials represent the most appropriate method to gather information regarding the comparative effectiveness and adverse events of a novel therapy as compared to the conventional treatment, in order to inform pragmatic treatment decisions. In this regard, *n*-of-1 trials retain promise for the advancement of precision medicine (81) and precision nutrition (14, 63). We argue that the only caveat in relation to the small number of participants included herein might stem from the high individual heterogeneity and the subsequent inability to pool findings to perform group statistical analyses with respect to the two intervention cycles.

Regarding the lack of repetition of the AB intervention phases, the AB quasi-experimental design is considered as the most appropriate in defining the most effective, adverse-event-free therapy for each patient (18, 82). On the other hand, repetitions of the AB phases in a sequential manner is to *n*-of-1 trials, what sample size is for parallel arm randomized controlled trials (20) and can defend against random error. Nevertheless, the simple quasi-experimental design employed herein allows for the direct comparison of treatments A (conventional MNT) and B (genotype-guided MNT) and protects against several forms of systematic error (including history, testing, regression to the mean) (20, 83).

Finally, an additional limitation involves the lack of recorded data regarding the patients' perspective with respect to the treatments. Future studies based on the proof-of-principle results presented herein will address these issues.

Through these limited in number, yet carefully designed trials and investigated cases, we have herein addressed a major gap in linking the observational nature of gene-diet interactions to an effective step-by-step process required for the implementation of precision nutrition interventions (14, 84). Supplementary Figure 2 outlines the strategy followed in the present case series, based on the nutrition care process (NCP) model that may serve as a guide for the implementation of innovative genotype-directed MNTs in T2DM as well as other polygenic, multifactorial pathologies. Overall, the present series of *n*-of-1 superiority trials indicates that precision nutrition is both feasible and effective in adults with pre-diabetes and T2DM. Given the worldwide prevalence of T2DM, we anticipate that this approach may assist nutritionists in providing more personalized nutritional recommendations, enabling patient adherence and improved health outcomes.

## Data Availability Statement

The raw data supporting the conclusions of this article will be made available by the authors, without undue reservation.

## Ethics Statement

The studies involving human participants were reviewed and approved by Bioethics Committee of the National and Kapodistrian University of Athens Medical School. The patients/participants provided their written informed consent to participate in this study. Written informed consent was obtained from the individual(s) for the publication of any potentially identifiable images or data included in this article.

## Author Contributions

KG and AE: conceptualization, methodology, and resources. KG: assays. EL and KG: data collection. KG, AE, and MG: data curation. DS, EL, and DG: writing—review and editing. AE: visualization and project administration. All authors have read and agreed to the published version of the manuscript. All authors contributed to the article and approved the submitted version.

## Conflict of Interest

Author KG was employed by Embiodiagnostics Biology Research Company. The remaining authors declare that the research was conducted in the absence of any commercial or financial relationships that could be construed as a potential conflict of interest.

## Publisher's Note

All claims expressed in this article are solely those of the authors and do not necessarily represent those of their affiliated organizations, or those of the publisher, the editors and the reviewers. Any product that may be evaluated in this article, or claim that may be made by its manufacturer, is not guaranteed or endorsed by the publisher.
