# Supplementary material for: Genetically-Guided Medical Nutrition Therapy in Type 2 Diabetes Mellitus and Pre-diabetes: A Series of n-of-1 Superiority Trials
Source: Front Nutr. 2022 Feb 21;9:772243. doi: 10.3389/fnut.2022.772243 (PMC8899711; doi:10.3389/fnut.2022.772243)
Supplement: Supplementary file 2 [file Data_Sheet_2.pdf]

Supplementary Material

Gkouskou KK, Grammatikopoulou MG, Lazou E, Sanoudou D, Goulis DG, Eliopoulos AG. Genetically-guided medical nutrition therapy in type 2 diabetes mellitus and prediabetes: a series of n-of-1 superiority trials. Frontiers in Nutrition 2022; 9: 772243.

Detailed methodology

Inclusion and exclusion criteria

Patients were eligible to participate provided that 1) they were adults; 2) with a diagnosis of type 2 diabetes mellitus (T2DM) or prediabetes based on the American Diabetes Association (ADA) standards of medical care (1); 3) willing to adhere to a lifestyle treatment including a personalized diet; 4) without any known malignancies, endocrine or metabolism-related diseases (e.g. renal disease) or genetic disorders affecting metabolism and nutritional status; 5) providing consent to the study participation; and 6) be able to communicate and understand the Greek language. Exclusion criteria involved 1) minors; 2) with comorbidities affecting metabolism; 3) unwilling to consent or adhere to lifestyle therapy; 4) unable to communicate in the Greek language.

Intervention and comparator

The energy requirements of all participants were estimated using the Mifflin-St. Jeor prediction equations (2,3) and the level of physical activity of each patient. The appropriate energy deficit level was calculated based on the estimated energy requirements (EER) and the goal for a 5% body weight (BW) loss, as suggested in the clinical practice guidelines for the treatment of prediabetes and T2DM (4). Two interventions were administered in a cross-over, non-randomized design. The procedure of each trial is detailed in Figure 1. Each patient was initiated with 8 weeks of conventional medical nutrition therapy (MNT), followed by 1 week of washout, and then a precision MNT intervention was applied for an additional 8-week period. For the conventional dietary intervention, the Problem, Etiology, Signs and Symptoms (PES) statement was structured for each patient, based on the nutrition care process (NCP) model, the revised standards of practice and the phenotypic characteristics of participants (5,6). Moreover, recommendations were based on the clinical practice guidelines of the ADA and Academy of Nutrition and Dietetics (AND), and as the latest evidence of higher hierarchy regarding prediabetes/T2DM MNT (7–9). With regard to exercise, this remained similar according to the patients' previous schedule unless otherwise indicated.

As for all cases response to the treatment was inadequate according to the predefined NCP outcomes within 8 weeks of the initial intervention, the genetic profile of each patient was used to guide a more personalized lifestyle intervention (diet and exercise), based on the available scientific evidence. No changes were made to the pharmacological treatment of participants. Baseline and end of treatment period measurements were recorded for each participant.

Adherence to the dietary treatment

Adherence to the treatment was assessed qualitatively, through telephone interviews by experienced dietitians (M.G.G, E.L). Previous 24h diet recalls were collected through these interviews regarding the diet of the participants. This is a retrospective short-term method for assessing dietary and food intake often performed through telephone interviews. Adherence to the oral nutrient supplementation (ONS) treatment (whenever applicable) was self-reported and exercise adherence was evaluated in the telephone interviews using specific questions assessing adherence and possible barriers.

Phenotypic profiling related to diabetes and weight status

Detailed phenotypic profiling of patients was performed by a multidisciplinary team of registered dietitians (E.L. and M.G.G) and endocrinologists (D.G.G), including a complete assessment of their dietary intake, physical activity levels and diabetes risk factors.

Anthropometric characteristics

BW and height of participants were measured during morning hours, by an experienced dietitian, with the use of a digital scale (SECA 813, SECA Group, Hamburg, Germany) and a wall-mounted stadiometer (SECA 216, SECA Group, Hamburg, Germany). At least duplicate measurements were taken. Body mass index (BMI) was calculated for each patient and weight status was defined according to the World Health Organization BMI thresholds (10). Waist circumference was measured at the iliac crest, with patients on a horizontal plane, and central obesity was diagnosed in those with a perimeter exceeding 94 cm (11).

Assay of blood markers

Morning fasting blood samples were collected from all patients for blood glucose and a lipidemic profile assay, including total cholesterol (TC), triglycerides (TG), high-density lipoprotein (HDL) and low-density lipoprotein (LDL) cholesterol. Moreover, glycosylated haemoglobin (HbA_{1c}) of all patients was assessed from a capillary fingerstick using a DCA 2000 analyzer (Bayer Diagnostics, Tarrytown, NY, USA). With respect to the availability of micronutrients, ferritin serum levels were assessed according to the International Committee for Standardization in Haematology (12) and 25-hydroxy vitamin D (25(OH)D) concentrations were evaluated via liquid chromatography-tandem mass spectrometry (LC-MS).

Blood pressure

Blood pressure (BP) was measured with patients in a seated, relaxed position, in the arm exhibiting the highest BP value, using an oscillometric device (Microlife Exact BP, Microlif AG, Widnau, Switzerland). Duplicate measurements were taken.

Outcomes of interest

For all participants, primary outcomes involved change (Δ) in BW (kg) and fasting plasma glucose (FPG) concentrations (mg/dL). Secondary outcomes were systolic and diastolic involved blood pressure (BP) (mm Hg), depressive symptoms (whenever applicable) and possible adverse events.

Depressive symptoms

For patients exhibiting depression traits, the Beck Depression Inventory (BDI) was used to assess possible improvement (13). The scale consists of 21 questions each with four possible answers in a Likert scale. Each answer provides a score of 0–3, giving a pooled total score that can range from 0 (complete lack of depression) to 63. The scale has been officially translated and validated in the Greek language and used in a plethora of research.

Genotyping and genetic scores for obesity, habitual coffee consumption, T2DM and elevated fasting plasma glucose

Buccal swabs were stored at 4°C and processed for DNA extraction within 24 hours using the Purelink Genomic DNA extraction mini kit (Invitrogen, Thermo Fisher Scientific, Waltham, Massachusetts, USA). Single nucleotide polymorphism (SNP) status was assessed on an Open Array Quant Studio 12X flex thermocycler (Applied Biosystems, Waltham, USA). The success rate of genotyping was 99%.

Genetic risk score (GRS) determining habitual coffee consumption (that affected recommendations for fat intake in the proposed diet) was calculated on the basis of 8 SNPs (*GCKR* rs1260326, *ABCG2* rs1481012, *AHR* rs4410790 and rs6968554, Max-like protein X interacting protein-like rs7800944, *POR* rs17685, *BDNF* rs6265, *CYP1A1* rs2470893, *CYP1A2* rs2472297, and *EFCAB5* rs9902453), with the use of a previously reported weighting method (14).

The GRS for T2DM (driving recommendations for the prescription of optimal protein intake) was calculated on the basis of the 31 SNPs *ADAMTS9* rs4607103, *ADCY5* rs11708067, *BCL11A* rs243021, *CDC123-CAMK1D* rs12779790, *CDKAL1* rs10946398, *CDKN2A/B* rs10811661, *CENTD2* rs1552224, *CHCHD9* rs13292136, *DGKB-TMEM195* rs2191349, *GCK* rs4607517, *GCKR* rs780094, *HMGA2* rs1531343, *HNF1A* rs7957197, *IGF2BP2* rs4402960, *IRS1* rs2943641, *JAZF1* rs864745, KCNQ1 rs231362, *KLF14* rs972283, *MTNR1B* rs10830963, *NOTCH2* rs10923931, *PPARG* rs1801282, *PRC1* rs8042680, *PROX1* rs340874, *RBMS1-ITGB6* rs7593730, *SLC30A8* rs13266634, *THADA* rs7578597, *TP53INP1* rs896854, *TSPAN8-LGR5* rs7961581, *WFS1* rs10010131, *ZBED3* rs4457053 and the *ZFAND6* rs11634397, with the use of a previously reported weighting method (15).

The GRS for elevated FPG (influencing recommendations for optimal fat intake in precision MNT pattern) was calculated on the basis of the 14 SNPs *PROX1* rs340874, *G6PC2* rs560887, *GCKR* rs780094, *SLC2A2* rs11920090, *ADCY5* rs11708067, *DGKB-TMEM195* rs2191349, *GCK* rs4607517, *GLIS3* rs7034200, A*DRA2A* rs10885122, *FADS1* rs174550, *CRY2* rs11605924, *MADD* rs7944584, *MTNR1B* rs10830963, and *C2CD4B* rs11071657, as suggested by Wang (16).

The GRS for DM (that was modified by the adoption of a Western dietary pattern) was calculated on the basis of 10 SNPs *HHEX* rs1111875, *CDKAL1* (rs7756992), *IGF2BP2* (rs4402960), *SLC30A8* rs13266634, *WFS1* (rs10010131), *CDKN2A/B* (rs564398 and rs10811661), *TCF7L2 (*rs12255372), *PPARG (*rs1801282), and *KCNJ11* (rs5219), as previously suggested (16).

The GRS for obesity was calculated as the weighted sum of risk alleles across 32 SNPs indicating the highest association with BMI and/or WHR (and affected recommendations for protein intake in proposed diet) (17–19).

Summary of the genetic association results that were used for the present study are available on the GIANT consortium website (20) for BMI (‘SNP_gwas_mc_merge_nogc.tbl.uniq.gz’) (21).

Furthermore, independent SNPs that could be used to modify each patient's dietary, lifestyle (including chrono-nutrition) and physical activity habits, were additionally genotyped. SNPs were studied in relation to the optimal macronutrient intake, fibre intake and diabetes status, including the *MTNR1B* rs1387153, *APOA5* rs662799, *CRY1* rs2287161, *PCSK7* rs236918, *GIPR* rs2287019, *IRS1* rs2943641 *PLIN-1* rs894160, *PPM1K* rs1440581, *DHCR7* rs12785878, *CLOCK* rs1801260 and rs4580704, *LEPR* rs3790433 *GCKR* rs780094, *TCF7L2* rs7903146 and rs12255372 (22). Studied SNPs that are relevant to micronutrient supplementation and DM included the *MTNR1B* rs10830963, *MTHFR* rs1801133 (23) and the *SLC30A8* rs13266634 (24). The *IRS1* rs2943641 was additionally assessed, as its effect on insulin is modified by circulating 25(OH)D concentrations (25). SNPs relevant to chrononutrition, lifestyle and physical activity choices included the *MTNR1B* rs10830963 (26,27), *SLC30A8* rs13266634 (28), as well as the *PPARG* rs1801282 (29).

Data monitoring

Three experts (A.G.E., D.G.G. and D.S.) comprised the data monitoring committee (DMC) according to the SPENT guidelines. The DMC had full access to the anonymously coded dataset of the study.

Statistical analyses

As each patient received a different, personalized treatment, this heterogeneity does not allow for a quantitative synthesis of data. Thus, for each patient, outcomes at the end of each intervention cycle were compared to the start of the intervention period (treatment-by-period interaction) (30). No missing data were apparent in the dataset.

Supplementary References

1. American Diabetes Association (ADA). 2. Classification and Diagnosis of Diabetes: Standards of Medical Care in Diabetes-2020. *Diabetes Care* (2020) **43**:S14–S31. doi:10.2337/dc20-S002

2. Madden AM, Mulrooney HM, Shah S. Estimation of energy expenditure using prediction equations in overweight and obese adults: a systematic review. *J Hum Nutr Diet* (2016) **29**:458–476. doi:10.1111/JHN.12355

3. Mifflin MD, St Jeor ST, Hill LA, Scott BJ, Daugherty SA, Koh YO. A new predictive equation for resting energy expenditure in healthy individuals. *Am J Clin Nutr* (1990) **51**:241–247. doi:10.1093/AJCN/51.2.241

4. Association AD. 8. Obesity Management for the Treatment of Type 2 Diabetes: Standards of Medical Care in Diabetes—2021. *Diabetes Care* (2021) **44**:S100–S110. doi:10.2337/DC21-S008

5. Noland D, Raj S. Academy of Nutrition and Dietetics: Revised 2019 Standards of Practice and Standards of Professional Performance for Registered Dietitian Nutritionists (Competent, Proficient, and Expert) in Nutrition in Integrative and Functional Medicine. *J Acad Nutr Diet* (2019) **119**:1019-1036.e47. doi:10.1016/j.jand.2019.02.010

6. Academy of Nutrition and Dietetics. *Abridged nutrition care process reference terminology*

(NCPT) manual : standardized terminology for the nutrition care process. 2017 editi. Chicago, IL: Academy of Nutrition and Dietetics (2017).

7. Zafar MI, Mills KE, Zheng J, Regmi A, Hu SQ, Gou L, Chen L-L. Low-glycemic index diets as an intervention for diabetes: a systematic review and meta-analysis. *Am J Clin Nutr* (2019) **110**:891–902. doi:10.1093/ajcn/nqz149

8. Evert AB, Dennison M, Gardner CD, Garvey WT, Lau KHK, MacLeod J, Mitri J, Pereira RF, Rawlings K, Robinson S, et al. Nutrition Therapy for Adults With Diabetes or Prediabetes: A Consensus Report. *Diabetes Care* (2019) **42**:731–754. doi:10.2337/DCI19-0014

9. Briggs Early K, Stanley K. Position of the Academy of Nutrition and Dietetics: The Role of Medical Nutrition Therapy and Registered Dietitian Nutritionists in the Prevention and Treatment of Prediabetes and Type 2 Diabetes. *J Acad Nutr Diet* (2018) **118**:343–353. doi:10.1016/j.jand.2017.11.021

10. World Health Organization. Obesity: preventing and managing the global epidemic. Report of a WHO consultation. *World Health Organ Tech Rep Ser* (2000) **894**:1–253.

11. Alberti KGMM, Zimmet P, Shaw J, IDF Epidemiology Task Force Consensus Group. The metabolic syndrome--a new worldwide definition. *Lancet (London, England)* (2005) **366**:1059–62. doi:10.1016/S0140-6736(05)67402-8

12. Proposed international standard of human ferritin for the serum ferritin assay. International Committee for Standardization in Haematology (Expert Panel on Iron). *Br J Haematol* (1985) **61**:61–63. doi:10.1111/J.1365-2141.1985.TB04060.X

13. Beck A, Steer R, Brown G. *Beck Depression Inventory*. 2nd ed. San Antonio, TX: Psychological Corporation (1996).

14. Han L, Ma W, Sun D, Heianza Y, Wang T, Zheng Y, Huang T, Duan D, Bray JGA, Champagne CM, et al. Genetic variation of habitual coffee consumption and glycemic changes in response to weight-loss diet intervention: the Preventing Overweight Using Novel Dietary Strategies (POUNDS LOST) trial. *Am J Clin Nutr* (2017) **106**:1321–1326. doi:10.3945/ajcn.117.156232

15. Huang T, Ley SH, Zheng Y, Wang T, Bray GA, Sacks FM, Qi L. Genetic susceptibility to diabetes and long-term improvement of insulin resistance and β cell function during weight loss: the Preventing Overweight Using Novel Dietary Strategies (POUNDS LOST) trial. *Am J Clin Nutr* (2016) **104**:198–204. doi:10.3945/ajcn.115.121186

16. Wang T, Huang T, Zheng Y, Rood J, Bray GA, Sacks FM, Qi L. Genetic variation of fasting glucose and changes in glycemia in response to 2-year weight-loss diet intervention: the POUNDS LOST trial. *Int J Obes (Lond)* (2016) **40**:1164–9. doi:10.1038/ijo.2016.41

17. Qi Q, Chu AY, Kang JH, Jensen MK, Curhan GC, Pasquale LR, Ridker PM, Hunter DJ, Willett WC, Rimm EB, et al. Sugar-Sweetened Beverages and Genetic Risk of Obesity. *N Engl J Med* (2012) **367**:1387–1396. doi:10.1056/NEJMoa1203039

18. Speliotes EK, Willer CJ, Berndt SI, Monda KL, Thorleifsson G, Jackson AU, Allen HL,

Lindgren CM, Luan J, Mägi R, et al. Association analyses of 249,796 individuals reveal 18 new loci associated with body mass index. *Nat Genet* (2010) **42**:937–948. doi:10.1038/ng.686

19. Chen Y, Zhou T, Sun D, Li X, Ma H, Liang Z, Heianza Y, Pei X, Bray GA, Sacks FM, et al. Distinct genetic subtypes of adiposity and glycemic changes in response to weight-loss diet intervention: the POUNDS Lost trial. *Eur J Nutr 2020 601* (2020) **60**:249–258. doi:10.1007/S00394-020-02244-X

20. GIANT consortium - Giant Consortium. Available at: https://portals.broadinstitute.org/collaboration/giant/index.php/Main_Page [Accessed December 15, 2021]

21. Locke AE, Kahali B, Berndt SI, Justice AE, Pers TH, Day FR, Powell C, Vedantam S, Buchkovich ML, Yang J, et al. Genetic studies of body mass index yield new insights for obesity biology. *Nature* (2015) **518**:197–206. doi:10.1038/NATURE14177

22. Gkouskou K, Lazou E, Skoufas E, Eliopoulos AG. Genetically Guided Mediterranean Diet for the Personalized Nutritional Management of Type 2 Diabetes Mellitus. *Nutrients* (2021) **13**: doi:10.3390/nu13020355

23. Kheradmand M, Maghbooli Z, Salemi S, Sanjari M. Associations of MTHFR C677T polymorphism with insulin resistance, results of NURSE Study (Nursing Unacquainted Related Stress Etiologies). *J Diabetes Metab Disord* (2017) **16**: doi:10.1186/S40200-017-0303-9

24. Gkouskou KK, Grammatikopoulou MG, Vlastos I, Sanoudou D, Eliopoulos AG. Genotype-guided dietary supplementation in precision nutrition. *Nutr Rev* (2020)nuaa132. doi:10.1093/NUTRIT/NUAA132

25. Zheng J-S, Parnell LD, Smith CE, Lee Y-C, Jamal-Allial A, Ma Y, Li D, Tucker KL, Ordovás JM, Lai C-Q. Circulating 25-Hydroxyvitamin D, IRS1 Variant rs2943641, and Insulin Resistance: Replication of a Gene–Nutrient Interaction in 4 Populations of Different Ancestries. *Clin Chem* (2014) **60**:186. doi:10.1373/CLINCHEM.2013.215251

26. Lopez-Minguez J, Saxena R, Bandín C, Scheer FA, Garaulet M. Late dinner impairs glucose tolerance in MTNR1B risk allele carriers: A randomized, cross-over study. *Clin Nutr* (2018) **37**:1133–1140. doi:10.1016/j.clnu.2017.04.003

27. Lane JM, Chang A-M, Bjonnes AC, Aeschbach D, Anderson C, Cade BE, Cain SW, Czeisler CA, Gharib SA, Gooley JJ, et al. Impact of Common Diabetes Risk Variant in MTNR1B on Sleep, Circadian, and Melatonin Physiology. *Diabetes* (2016) **65**:1741–51. doi:10.2337/db15-0999

28. Sprouse C, Gordish-Dressman H, Orkunoglu-Suer EF, Lipof JS, Moeckel-Cole S, Patel RR, Adham K, Larkin JS, Hubal MJ, Kearns AK, et al. SLC30A8 nonsynonymous variant is associated with recovery following exercise and skeletal muscle size and strength. *Diabetes* (2014) **63**:363–8. doi:10.2337/db13-1150

29. Ruchat S-M, Rankinen T, Weisnagel SJ, Rice T, Rao DC, Bergman RN, Bouchard C, Pérusse

L. Improvements in glucose homeostasis in response to regular exercise are influenced by the PPARG Pro12Ala variant: results from the HERITAGE Family Study. *Diabetologia* (2010) **53**:679–89. doi:10.1007/s00125-009-1630-2

30. Lichtenstein AH, Petersen K, Barger K, Hansen KE, Anderson CAM, Baer DJ, Lampe JW, Rasmussen H, Matthan NR. Perspective: Design and Conduct of Human Nutrition Randomized Controlled Trials. *Adv Nutr* (2021) **12**:4–20. doi:10.1093/advances/nmaa109
