# Supplementary material for: Genetically-Guided Medical Nutrition Therapy in Type 2 Diabetes Mellitus and Pre-diabetes: A Series of n-of-1 Superiority Trials
Source: Front Nutr. 2022 Feb 21;9:772243. doi: 10.3389/fnut.2022.772243 (PMC8899711; doi:10.3389/fnut.2022.772243)
Supplement: Supplementary file 3 [file Image_1.pdf]

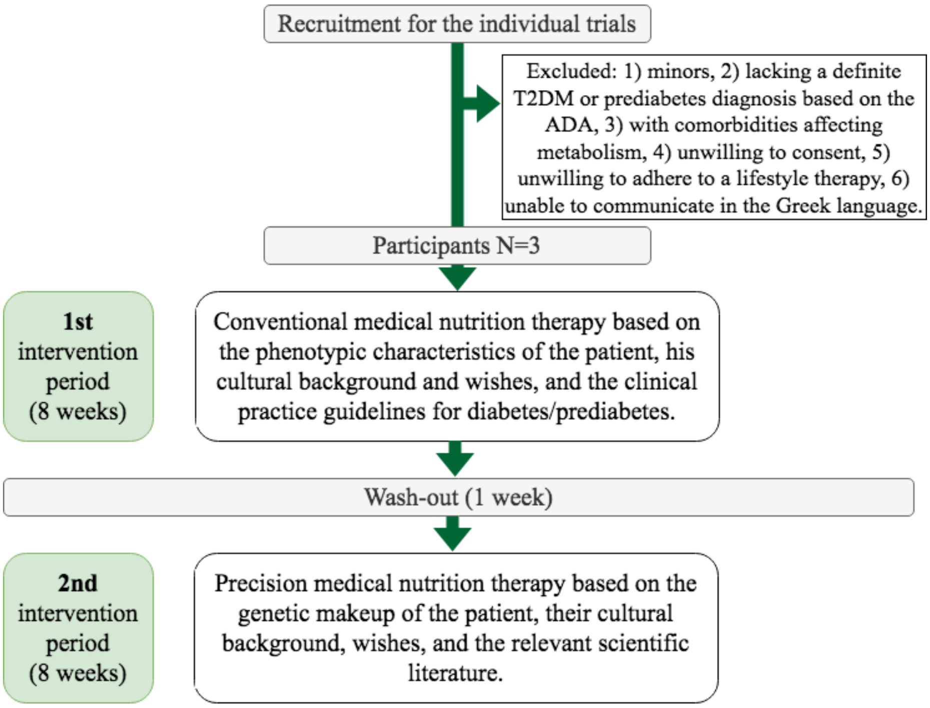


**Supplementary Figure 1.** Flowchart of the study process; *ADA: American Diabetes Association; T2DM: Type 2 diabetes mellitus.*
