# Supplementary material for: Genetically-Guided Medical Nutrition Therapy in Type 2 Diabetes Mellitus and Pre-diabetes: A Series of n-of-1 Superiority Trials
Source: Front Nutr. 2022 Feb 21;9:772243. doi: 10.3389/fnut.2022.772243 (PMC8899711; doi:10.3389/fnut.2022.772243)
Supplement: Supplementary file 4 [file Image_2.pdf]

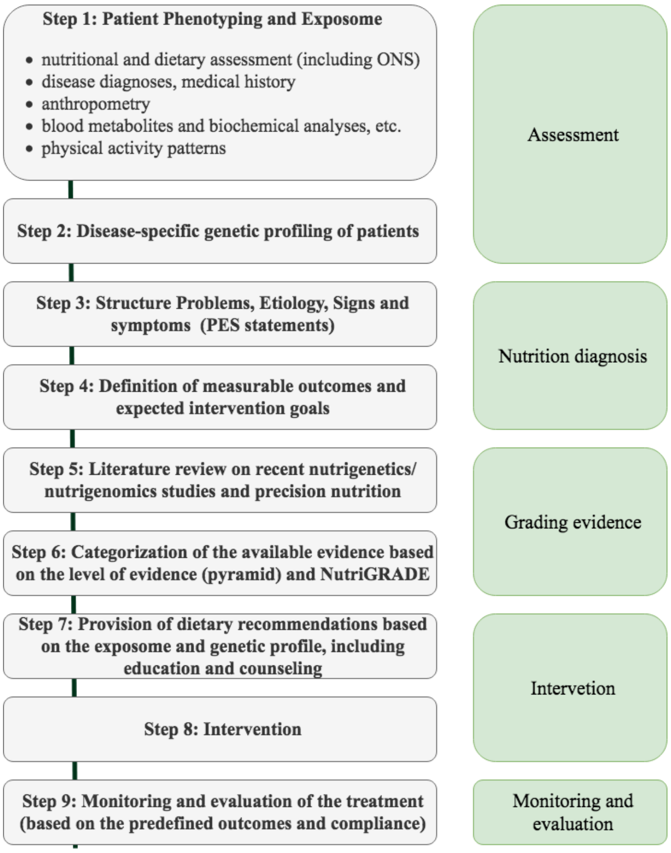

Supplementary Figure 2. Proposed roadmap for the implementation of precision nutrition interventions based on nutrigenetics and nutrigenomics data and the NCP model. *NCP: Nutrition care process; ONS: oral nutrient supplementation.*
