# Supplementary material for: Genetically-Guided Medical Nutrition Therapy in Type 2 Diabetes Mellitus and Pre-diabetes: A Series of n-of-1 Superiority Trials
Source: Front Nutr. 2022 Feb 21;9:772243. doi: 10.3389/fnut.2022.772243 (PMC8899711; doi:10.3389/fnut.2022.772243)
Supplement: Supplementary file 5 [file Table_1.pdf]

Supplementary Table 1. PICO strategy of the study's research question

PICO	PICO components
**P**opulation	Patients with prediabetes or type 2 diabetes mellitus
**I**ntervention	Precision medical nutrition therapy
**C**omparison	Conventional medical nutrition therapy
**O**utcome(s)	Body weight, systolic and diastolic blood pressure, glycosylated haemoglobin (HbA_{1c}), fasting plasma glucose
